# The Correlation between Serum Zinc Level and Liver Histology in Non-Alcoholic Steatohepatitis

**DOI:** 10.30699/IJP.14.1.17

**Published:** 2018-12-27

**Authors:** Farid Kosari, Raika Jamali, Tayeb Ramim, Ebrahim Mosavi Jahan Abad

**Affiliations:** 1 *Associate Professor, Dept of Pathology, Sina Hospital, Tehran University of Medical Sciences, Tehran, Iran*; 2 *Associate Professor, Research Development Center of Sina Hospital, Digestive Disease Research Institute, NAFLD Research Center, Tehran University of Medical Sciences, Tehran, Iran*; 3 *Researcher, Sina Trauma and Surgery Research Center, Tehran University of Medical Sciences, Tehran, Iran*; 4 *Resident, Dept of Internal Medicine, Sina Hospital, Tehran University of Medical Sciences, Tehran, Iran*

**Keywords:** Non-alcoholic Fatty Liver Disease Zinc, Oxidative stress, Lobular inflammation, Fibrosis

## Abstract

**Background & Objective:**

The aim of this present study was to assess the relationship between serum zinc levels and liver histopathological findings in non-alcoholic steatohepatitis (NASH) patients.

**Methods:**

This case-control study was performed in consecutively selected NASH patients who had been referred to a general hospital. The control group consisted of age and sex-matched individuals with normal physical examinations, laboratory findings, and liver ultrasounds. Serum zinc level was measured using atomic absorption spectrophotometry. Liver histopathological findings were determined based on non-alcoholic fatty liver activity score.

**Results:**

A cohort of eighty biopsy-proven NASH patients and eighty controls were enrolled in the study. The mean serum zinc level was significantly lower in the NASH group compared with the controls. The mean serum zinc concen- tration was significantly lower in moderate and severe lobular inflammation groups than the mild group. After multiple adjustments for potential contributing variables, serum zinc level was associated with the severity of lobular inflam- mation. Nonetheless, it was not associated with liver steatosis and fibrosis. A serum zinc value of 89 (µg/dl) yielded a sensitivity and specificity of 93% and 86%, respectively, characterizing patients with lobular inflammation of less than two inflammatory foci per high-power field (HPF) from more advanced groups. Furthermore, a value of 79.55 (µg/dl) yielded a sensitivity and specificity of 87% and 100%, respectively, distinguishing those with a lobular inflammation grade of less than four foci per HPF from more advanced cases.

**Conclusion:**

Serum zinc level might be associated with the severity of lobular inflammation in NASH.

## Introduction

Non-alcoholic fatty liver disease (NAFLD) includes a wide range of disorders that consist of simple fatty infiltra- tion, steatohepatitis (NASH), and end-stage liver disease (cirrhosis) (1). It is estimated that the incidence of NASH in the Iranian general population is approximately 2% (2). Considering the obesity epidemic and the rapid increase of the burden of metabolic syndrome, NASH has become a major healthcare concern.

Liver biopsy is the gold standard method for the diagnosis and estimation of liver cell damage in NASH (3). However, its potential complications have limited its application in common clinical practice. To overcome this shortcoming, a variety of serum biomarkers have been evaluated to substitute for liver biopsy (4-6). It is worthnoting that the fluctuations in the serum concentrations of candidate markers during the course of NASH have limited their accuracy. Thus, it seems reasonable to explore more stable serum biomarkers, in order to find a precise predictor of liver histopathogy. The role of the enzymes catalyzing the process of scavenging free radical oxygen species, including superoxide dismutase (SOD) type one, in the NASH, is well-studied (7-10). However, there is still a paucity of literature regarding the association of SOD and hepatocyte inflammation and necrosis. Considering that zinc is the main element present in SOD type one, we proposed that its serum concentration might be a predictor of liver cell damage.

The aims of this current research were to 1) compare serum zinc levels between NASH patients and controls, and 2) evaluate its association with liver histopathological findings.

## Material and Methods

Patient Enrollment

This case-control study was conducted in the gastroenterology clinic of a general hospital affiliated with the School of Medicine at the Tehran University of Medical Sciences. Individuals with persistently elevated serum aminotransferase levels (>40 IU/L) were consecutively included in the study from 2013 to 2015 (11). Those with cirrhosis, a history of taking hepatotoxic medications during the previous 6 months, alcohol, viral hepatitis, auto- immune hepatitis, Wilson’s disease, and hemochromatosis were excluded from the study. Furthermore, subjects with conditions that could influence the serum zinc concentrations, including renal failure (glomerular filtration rate <60 mL/minute), consumption of specific medications (including diuretics, penicillamine, or ACE Inhibi- tors), chronic diarrhea, and signs of malabsorption, were excluded (Phase 1). Thereafter, all subjects underwent hepatic ultrasounds, and individuals who did not fulfill the criteria of fatty liver were excluded (Phase 2). Liver biopsy was conducted in the remaining cases to confirm the diagnosis of NASH, and to assess severity of fatty liver disease based on “NAFLD activity Score” (NAS; Phase 3).

The control group consisted of subjects with normal physical examinations, serum level of liver enzymes, and liver ultrasound examinations. An epidemiologist who was masked to the clinical and paraclinical data of the participants, matched the cases and controls with regards to age and sex by applying the block matching method.

Ethical Considerations

The study was conducted according to ethical standards for human experimentation (Helsinki Declaration). The study protocol was approved by the Ethics Committee of the Tehran University of Medical Sciences (Reg- istration number: 9011160055). The participants were informed about the aim and method of the study. They entered the study after completing written informed consent forms.

Sample Size Calculation

The sample size was calculated as eighty, assuming the mean prevalence of NAFLD (P = 28%, α = 0.05, z =

1.96, and d = 0.12), according to the previous studies (3, 12).

Laboratory Investigations

At the time of the liver biopsy, fasting blood samples (5cc) were taken. The serum was separated and stored at

-80•C pending further evaluation. The serum zinc level was measured using atomic absorption spectrophotometry; fasting blood glucose, insulin, lipid profiles and liver function tests were measured as previously described (12, 13), and azll laboratory measurements were performed twice in the standard environment according to the manufacturer’s instructions. The calculated coefficient of variation was less than five percent in current experimentations.

Liver Biopsy

A percutaneous liver biopsy was carried out with a true cut needle (G14). A biopsy section more than ten millimeters in length, or with at least five portal tracts in microscopic view, was considered acceptable for his- topathologic examination. Hematoxylin and Eosin staining was performed to assess necroinflammation, and Masson’s Trichrome staining was done to evaluate liver fibrosis. A single expert gastrointestinal pathologist, who was blinded to patient data interpreted liver biopsy slides. The severity of liver steatosis, lobular inflamma- tion and fibrosis, was defined based on the “non-alcoholic fatty liver activity score (NAS)” (14). Twenty-five percent of randomly selected slides were reviewed again by the same pathologist at the end of project to discover intra-observer variability. The agreement of histopathological findings was ninety-five percent in the reviewed slides. Randomization of the pathology slides was done by the clinical epidemiologist who was masked to the participants’ clinical and paraclinical information.

Statistical Analysis

The distribution of data was assessed by the Kolmogorov-Smirnov test. Continuous variables were reported as mean (± standard deviation). Comparisons of mean age, clinical data, serum level of liver enzymes, metabolic profile, and serum zinc concentration between NASH and the controls were performed using an independent t- test. The correlation between liver histology findings and serum zinc values was assessed by the General Linear Model. To adjust the effect of potentially confounding factors regarding liver histology findings, we considered the BMI, liver function tests, and metabolic profile as covariates. The pair-wise comparisons of estimated marginal means of serum zinc level between liver histology groups were performed using the Bonferroni post-hoc test.

Patients with 5%-33%, 33%-66%, >66% fatty liver were labeled as mild, moderate and severe groups, respectively. Those with less than two foci per high-power field (HPF), two - four foci per HPF, and more than four foci per HPF were classified as mild, moderate, and severe groups respectively. Subjects with per sinusoidal, periportal, and bridging fibrosis were categorized as mild, moderate, and severe groups respectively.

Receiver operating characteristic (ROC) analysis was performed to find out the cut-off values of serum zinc in order to discriminate patients with a lobular inflammation of less than two foci per HPF from those with more advanced inflammation. The mentioned analysis also determined the threshold values of serum zinc, to distinguish patients with a lobular inflammation of less than four foci per HPF from more advanced ones. The best cutoff value was measured in a way that would net the highest sensitivity and specificity. The Area Under Curve (AUC) with 95% confidence interval was reported.

A two-sided p value of 0.05 or less was considered statistically significant in all analyses. A medical statistician performed the statistical calculations using IBM SPSS Statistics version 21 (IBM Corporation, Somers, NY, USA).

**Table 1:** The characteristics of the study population

**Variables**		**Non-alcoholic steatohepatitis**	**Control Group**	***P***
Age (years)		Age (years)	Age (years)	Age (years)
Waist circumference (cm)		3.56±101.09	4.33±98.26	0.01
Body mass index (Kg/m2)		5.77±32.88	6.73±30.23	0.06
Gender	Male (n)	48	45	0.69
	Female (n)	32	35	
Metabolic	Positive (n)	49	27	<0.01
	Negative (n)	31	53	
Aspartate aminotransferase (IU/L)		16.15±67.88	14.56±27.12	0.03
Alanine aminotransferase (IU/L)		12.33±56.35	10.43±26.22	<0.01
Alkaline phosphatase(IU/L)		190.60±47.13	187.80±33.23	0.38
Gamma-glutamyl transpeptidase (IU/L)		23.55±53.22	52.97±25.60	0.14
Fasting blood glucose (mg/dl)		97.148.12±	89.237.25±	0.001
Triglyceride (mg/dl)		156.0740.26±	98.0439.39±	<0.001
Cholesterol (mg/dl)		176.4834.17±	162.3344.36±	0.36
Low density lipoprotein (mg/dl)		102.5818.12±	98.35±16.57	0.81
High density lipoprotein (mg/dl)		46.4910.60±	52.139.06±	0.34
Insulin (mU/L)		16.25±10.42	8.40±5.09	<0.001
Zinc (µg/dl)		89.42±11.56	101.34±5.70	<0.001

Cm: centimeter, Kg: kilogram, M: meter, IU: international unit, L: liter, mg: milligram, dl: deciliter, mU: mili unit, µg: microgram.

Data presented as mean ± standard deviation, unless otherwise specified.

**Figure 1:**
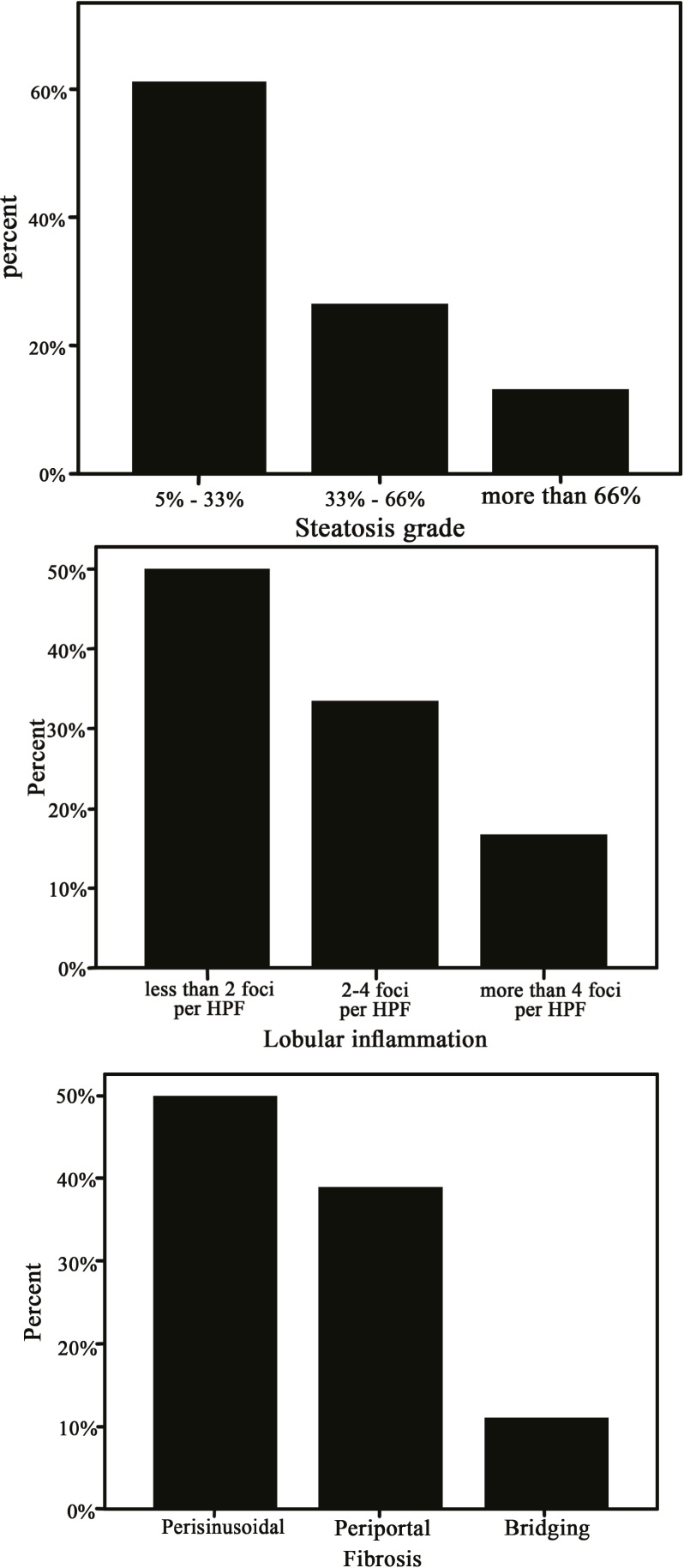
The frequency of liver histology findings in subjects with non-alcoholic steatohepatitis. Steatosis grades (Top), in- flammation grades based on foci of lobular inflammation in high power field (HPF) of microscopic view (Middle), and fibrosis stages (Bottom)

## Results

A total of eighty cases and eighty controls were enrolled in the study. The characteristics of the studied population are presented in the mean serum zinc level was significantly lower in the NASH group than the controls. The percent frequencies of the liver histopathological findings with regards to steatosis grade, lobular inflammation grade, and fibrosis stage are demonstrated in Fig. 1.

The comparisons of mean serum zinc levels between liver steatosis, lobular inflammation, and fibrosis groups are shown in Table 2. The mean serum zinc concentration was significantly lower in moderate and severe lobular inflammation groups than the mild group. No significant difference was identified in the zinc serum levels between subjects with various liver steatosis and fibrosis.

**Figure 2:**
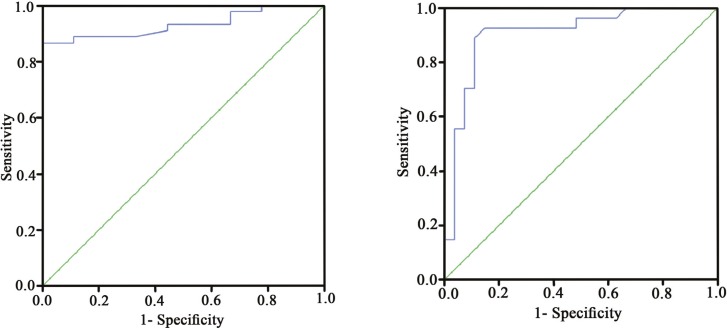
Receiver operating characteristic (ROC) analysis to determine threshold values of serum zinc for differentiating lobular inflammation severity

A serum zinc value of 89 (µg/dl) yielded a sensitivity and specificity of 93% and 86% respectively, separating patients with a lobular inflammation grade of less than two foci per high power field (HPF) from more advanced groups. Meanwhile, the value of 79.55 (µg/dl) yielded a sensitivity and specificity of 87% and 100% respectively, distinguishing those with lobular inflammation grade of less than four foci per HPF from more advanced cases (Figure. 2).

**Table 2:** Serum zinc level in subjects based on liver steatosis, lobular inflammation, and fibrosis groups

**Histopathological finding**	**Severity**	**Serum zinc level**	**P (ANOVA)**
Steatosis	Mild grade (5%-33%)	91.40±10.54	1.00*
	Moderate (33%-66%)	89.24±13.05	0.58**
	Severe (>66%)	79.68±10.16	0.51***
Lobular inflammation	Mild (1-2 foci per HPF)	97.44±8.59	<0.01*
	Moderate (2-4 foci per HPF)	84.41±8.21	<0.01**
	Severe (>4 foci per HPF)	74.95±3.52	0.10***
Fibrosis	Mild (perisinusoidal)	91.76±10.17	0.19*
	Moderate (periportal)	86.11±13.84	1.00**
	Severe (bridging)	89.80±8.72	0.47***

Data presented as mean ± standard deviation HPF: High power field

* Indicates the comparison between mild and moderate groups

** Indicates the comparison between mild and severe groups

*** Indicates the comparison between moderate and severe groups

A: ROC curve of serum zinc levels to differentiate lobular inflammation grade of less than two foci per high power field (HPF) from more ad- vanced grades of lobular inflammation [AUC: 91% (95% Confidence interval: 82%-99%)]; B: ROC curve of serum zinc levels to differentiate lobular inflammation grade of less than four foci per HPF from more advanced grades of inflammation. [AUC: 93% (95% Confidence interval:87%-99%)] AUC: Area under curve.

## Discussion

Zinc exists in the SOD. This enzyme performs key antioxidant activity in the course of hepatocyte inflamma- tion. Considering the possible role of zinc in the course of steatohepatitis, we compared serum zinc concentra- tions in the NASH with healthy subjects. The mean serum zinc level was significantly lower in the NASH group than the controls in our study. This finding is in line with a previous study that showed a lower amount of serum zinc alpha 2 glycoprotein in NAFLD patients than healthy controls (15). The mentioned study recognized serum zinc alpha 2 glycoprotein as an independent predictor of metabolic syndrome components in NAFLD (15). In parallel to our results, another research demonstrated decreased serum zinc level in individuals with viral hepa- titis compared with the reference group (16). The study suggested that deficiency in serum zinc causes reduced antioxidant activity, increased lipid peroxidation, and liver cell injury in chronic hepatitis (16).

The result of our project is comparable to previous investigations that reported lower serum SOD in NASH than the controls (7, 8). This data is also in accordance with a former report that indicates that an increase in SOD activity ameliorates lipid peroxidation and oxidative stress in methionine-choline deficient diet-induced hepatic steatosis mice (9). Meanwhile, another investigation demonstrated higher serum manganese containing SOD in NAFLD patients with more advanced lobular inflammation and fibrosis (10).

The accumulation of fatty acids in hepatocytes triggers the release of inflammatory cytokines via augmenta- tion of oxidative stress. The balance between the pro and anti-oxidants determines the course of the subsequent inflammatory response and concomitant healing process (fibrosis). SOD is an important antioxidant system that protects hepatocytes against oxidative stress. It is classified as three major types according to the binding metal cofactor. Type one, which is located in the cytoplasm, binds both copper and zinc; type two exists in the mitochondria and binds either iron or manganese; and type three is extracellular and binds nickel. Evaluating the different types of SOD in the above-mentioned experiments might interpret the controversy in their results.

To the best of our knowledge, this is the first inspection that assesses the correlation of serum zinc concentra- tion with the severity of liver steatosis, inflammation, and fibrosis in NAFLD. Controlling for potential third variables (including BMI, liver function tests, and metabolic profile), the mean serum zinc value was signifi- cantly lower in moderate and severe lobular inflammation groups than the mild group; however, no significant difference was observed with regard to liver steatosis and fibrosis. Furthermore, we defined the serum zinc threshold values for discriminating patients with a lobular inflammation grade of less than two foci per HPF from more advanced groups, and those with lobular inflammation grade of four foci per HPF from more ad- vanced groups. ROC curves showed a great accuracy with regards to the high Area Under Curve (AUC) of both measured threshold values of serum zinc.

One of the strengths of this study lay in evaluating a cohort of biopsy-proven NASH. We considered nearly all the possible factors that might affect liver histopathology in NASH. Meanwhile, possible factors that could affect the serum zinc concentration were noted and patients with renal failure, chronic diarrhea, and signs of malabsorption were excluded. Moreover, the histopathological findings were defined based on the “non-alcoholic fatty liver activity score”. This score has an appropriate accuracy and reliability in diagnosing NASH and determining the severity of its histopathological involvement. To omit the inter-observer variability, one expert gastroenterology pathologist reviewed the liver biopsy slides. In addition, intra-observer variability was assessed by reviewing randomly selected slides for the second time by the pathologist, which showed a significant agreement (ninety-five percent) regarding histopathogy findings.

Our study has several limitations that need to be considered. First, no causal relationship between serum zinc level and hepatic inflammation can be drawn due to the cross sectional nature of the study. Another limitation is the possibility of sampling bias. The mean age of cases was thirty-six years and cirrhotic patients were excluded from this research. Thus, our study subjects were relatively young, and in the early stage of NASH; therefore, the long-term consequences of NASH might not have been evident in our study subjects yet. The current results might not be generalized to all subjects with NASH.

It is wise to measure serum zinc and SOD levels concomitantly to define their role in hepatocyte inflammation in future studies. The causal relationship between serum zinc level and hepatic inflammation should be further clarified by large cohort studies. Also, if casual association is established, conducting a clinical trial to evalu- ate the effect of zinc supplements on the severity of hepatic inflammation in NASH is recommended. It seems reasonable to determine the association of zinc and liver histology findings in patients with advanced diseases including cirrhotic and pre-transplantation states.

## Conclusion

This case-control study evaluated the association between serum zinc level and NASH. Consecutively-selected subjects with NASH who had been admitted to a gastroenterology clinic were enrolled in the study. Controls consisted of age and sex-matched subjects who had normal physical examinations, liver function tests, and liver ultrasound examinations. Serum zinc concentration was significantly lower in NASH patients than controls. After multiple adjustments in order to control the factors related to NASH and serum zinc level, the association between serum zinc level and liver histological findings remained significant. The mean serum zinc level was significantly lower in moderate and severe lobular inflammation groups than in the mild group. Finally, the best cut-off values of serum zinc were determined to detect the severity of liver lobular inflammation.
